# Tackling of Renal Carcinogenesis in Wistar Rats by *Silybum marianum* Total Extract, Silymarin, and Silibinin *via* Modulation of Oxidative Stress, Apoptosis, Nrf2, PPAR*γ*, NF-*κ*B, and PI3K/Akt Signaling Pathways

**DOI:** 10.1155/2021/7665169

**Published:** 2021-09-30

**Authors:** Nour Y. S. Yassin, Sameh F. AbouZid, Asmaa M. El-Kalaawy, Tarek M. Ali, Basem H. Elesawy, Osama M. Ahmed

**Affiliations:** ^1^Physiology Division, Zoology Department, Faculty of Science, Beni-Suef University, P.O. Box 62521, Beni-Suef, Egypt; ^2^Department of Pharmacognosy, Faculty of Pharmacy, Beni-Suef University, Beni-Suef, Egypt; ^3^Department of Pharmacology, Faculty of Medicine, Beni-Suef University, Beni-Suef, Egypt; ^4^Department of Physiology, College of Medicine, Taif University, P.O. Box 11099, Taif 21944, Saudi Arabia; ^5^Department of Pathology, College of Medicine, Taif University, P.O. Box 11099, Taif 21944, Saudi Arabia

## Abstract

The present work was designed to assess the efficacy of *Silybum marianum* total extract (STE), silymarin (Sm), and silibinin (Sb) against experimentally induced renal carcinogenesis in male Wistar rats and their roles in regulating oxidative stress, inflammation, apoptosis, and carcinogenesis. The diethylnitrosamine (DEN)/2-acetylaminofluorene (AAF)/carbon tetrachloride (CCl_4_)-administered rats were orally treated with STE (200 mg/kg b.w.), Sm (150 mg/kg b.w.), and Sb (5 mg/kg b.w.) every other day either from the 1^st^ week or from the 16^th^ week of carcinogen administration to the end of 25^th^ week. The treatments with STE, Sm, and Sb attenuated markers of toxicity in serum, decreased kidney lipid peroxidation (LPO), and significantly reinforced the renal antioxidant armory. The biochemical results were further confirmed by the histopathological alterations. The treatments also led to suppression of proinflammatory mediators such as NF-*κβ*, p65, I*κβα*, and IL-6 in association with inhibition of the PI3K/Akt pathway. Furthermore, they activated the expressions of PPARs, Nrf2, and IL-4 in addition to downregulation of apoptotic proteins p53 and caspase-3 and upregulation of antiapoptotic mediator Bcl-2. The obtained data supply potent proof for the efficacy of STE, Sm, and Sb to counteract renal carcinogenesis *via* alteration of varied molecular pathways.

## 1. Introduction

Kidney cancer is one of the most common malignant tumors of the genitourinary system, and it is among the cancer types that have the highest growth rate in all age and racial groups worldwide [1]. It has been stated that it is the most resistant malignancy, responding to conventional therapies either very little or not at all [2]. Despite the discovery of many novel chemotherapeutic drugs in the last decade as well as advances in the understanding of molecular pathways implicated in the development of kidney carcinogenesis, the disease remains incurable and fatal [3]. Several risk factors are claimed to predispose to kidney cancer involving chronic renal disease, hereditary syndrome, hypertension, obesity, cigarette smoking, long-term dialysis, genetic susceptibility, and maybe the result of exposure to different environmental toxicants [4].

Oxidative stress is defined as an imbalance between the generation of reactive oxygen species (ROS) and the biological system's ability to counteract the effects of reactive free radicals or repair oxidative damage [5]. High amounts of ROS can also compromise the antioxidant defense system resulting in DNA, protein, and lipid damage [6]. The diminished effectiveness of the antioxidant defense system may aggravate oxidative damage [7]. ROS such as superoxides, peroxides, and hydroxyl radicals can be produced by mitochondria or extramitochondrial NAD (P)H oxidase (Nox) system [8, 9]. Oxidative stress is a major cause of various renal diseases that stimulates progression from acute to chronic damage and the development of kidney cancer [10].

Accumulating evidence indicates that tumorigenesis, rather than occurring spontaneously, appears to be linked to chronic inflammation and immune dysfunction [11, 12]. It has been proved that inflammation causes chromosomal instability, a proliferation of cancer cells, and stimulation of angiogenesis and tissue remodeling [13, 14]. Oxidative stress triggers the activation of nuclear factor-kappa B (NF-*κ*B), exacerbating inflammation *via* inhibition of peroxisome-activated receptors (PPARs) and nuclear factor erythroid 2-related factor 2 (Nrf2) and anti-inflammatory interleukins [15–18].

Nrf2 controls the transcription of several antioxidant genes that maintain detoxification genes and cellular homeostasis in check, allowing toxins and carcinogens to be removed before they cause damage [19]. Nrf2 is known to be expressed in all cell types; however, it translocates into the nucleus during elevated cellular stress and stimulates the transcription of target genes encoding proteins related to xenobiotic efflux, redox regulation, iron metabolism, protein homeostasis, resistance to apoptosis, and repairing DNA [20]. Consequently, Nrf2 is thought to promote several anti-inflammatory effects involving inhibition of NF-*κ*B activity, reduction of the expressions of several inflammatory mediators involving chemokines, cytokines, adhesion molecules, MMP-9, iNOS, and COX-2 [21–23], and interfering with interleukin production [24] and directly enhances PPAR*γ* expression [25]. A previous study has examined the strategy of utilizing Nrf2 promoting compounds to mitigate ROS to inhibit the progression of renal diseases [26].

The PI3K/Akt signaling pathway is the most commonly activated pathway implicated in the oncogenesis of several cancers [27]. Activation of this pathway causes phosphorylation of additional proteins that regulate cell cycle entry, cell proliferation, and carcinogenesis [28, 29]. ROS can act directly on Akt or its downstream targets such as p53 and FOXO or indirectly through modulators such as GSK3, PTEN, Src, and others [30]. Genetic alterations in the PI3K/Akt pathway are common in kidney cancer [31, 32]. Therefore, PI3K/Akt signaling represents a wide window for developing targeted therapy.

Earlier studies investigated the roles of various dietary and natural antioxidants against damage caused by oxidative stress affecting glomerular and tubular functions [33, 34]. Silymarin (Sm) and silibinin (Sb) are investigated to exert strong antineoplastic effects in both *in vitro* and *in vivo* models of cancer, including cancers of the prostate, bladder, skin, colon, lung, breast, and kidney [35–41]. During Sm and Sb treatment, several mechanisms have been applied, such as DNA repair, cell cycle arrest, inhibition of growth and proliferation in addition to antiangiogenic effects, and blockage of invasion and metastasis [42, 43].

In conductance with the previous literature, the purpose of this study was to evaluate the potential preventive and therapeutic effects of STE, Sm, and Sb on diethylnitrosamine (DEN)/2-acetylaminofluorene (AAF)/carbon tetrachloride (CCl_4_)-mediated renal carcinogenesis in addition to assessing their preventive mechanisms on apoptosis, Nrf2, PPAR*γ*, NF-*κ*B, and PI3K/Akt signaling pathways.

## 2. Materials and Methods

### 2.1. Chemicals

DEN, AAF, CCl_4_, and Sb were obtained from Sigma chemicals company (St. Louis, MO, USA). Assay kits for urea, creatinine, and uric acid were purchased from Biosystem (Spain). Antibodies for p-PI3K and *β*-actin were obtained from Santa Cruz Biotechnology (USA). t-PI3K, t-Akt, p-Akt, t-p65, p-p65, t-I*κ*B*α*, and p-I*κ*B*α* antibodies were purchased from Cell Signaling Technology, Inc. (USA). Cleaved-caspase-3 antibody was obtained from Merck Millipore (Germany). Other analytical grade reagents and chemicals were purchased from local suppliers.

### 2.2. Preparation of Silymarin and *Silybum marianum* Total Extract

The fruits of *Silybum marianum* were obtained from a local vendor and used to extract Sm and STE using a Soxhlet apparatus. The extraction was performed in two steps: first, the fruits were defatted for 6 hrs using petroleum ether, and then, these defatted fruits were soaked in methanol for 5 hrs to obtain pure Sm. Each extract was evaporated under reduced pressure at a temperature not exceeding 40°C. Finally, two extracts were obtained: the first extract is about a pure Sm and the second extract is STE which is about Sm and the petroleum ether extract. Quantitative nuclear magnetic resonance (NMR) was used for quantitative estimation of flavonolignans in the extracts used in assessing biological activities. This technique is a nondestructive process, and the samples need only a few steps to be prepared. It is easily automated and supplies both quantitative and qualitative analyte measurements without the use of chromatographic separation or extra analytical instrumentation. Sb was used as a reference standard compound. The calibration curve was done by plotting integration values versus the molar concentration. A linear relationship of the standard curve was used to obtain the regression equation [*y* = 0.5699*x* − 0.733 (*R*^2^ = 0.9987)] [44].

### 2.3. Animals

Eighty adult male Wistar rats (100–120 g body weight (b.w.)) were obtained from the Egyptian Organization for Biological Products and Vaccines (Helwan Station, Cairo, Egypt). Rats were kept under pathogen-free conditions. All procedures were carried out according to the rules of the animal care and use guidelines stated by the Experimental Animal Ethics Committee of Faculty of Science, Beni-Suef University, Egypt (Ethical Approval Number: BSU/FS/2017/6).

### 2.4. Experimental Design

**Figure 1:**
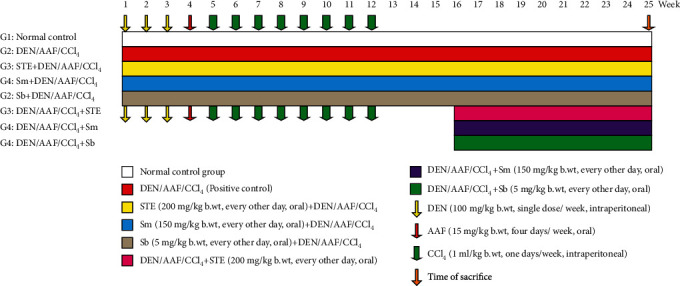
Experimental design.

After one week of adaptation, the rats were divided into eight groups each with 10 rats. Group I acted as a normal control group. Rats of the other seven groups were intraperitoneally injected with DEN (100 mg/kg b.w.) once a week for three successive weeks, followed by oral administration of AAF (15 mg/kg b.w.) four times a week for one week only. Starting from the 5^th^ week, all rats were injected with CCl_4_ (1 mL/kg b.w.) intraperitoneally until the 12^th^ week of the experiment [45]. Group II served as the DEN/AAF/CCl_4_ control group. Groups III, IV, and V received DEN/AAF/CCl_4_ like group II and were orally concurrently treated with STE (200 mg/kg b.w.) [46], Sm (150 mg/kg b.w.) [47], and Sb (5 mg/kg b.w.) [48], every other day for 25 weeks. Groups VI, VII, and VIII received DEN/AAF/CCl_4_ like group II, and after 16 weeks, they were treated with STE (200 mg/kg b.w.) [46], Sm (150 mg/kg b.w.) [47], and Sb (5 mg/kg b.w.) [48], every other day till the end of the 25^th^ week of the experiment (Figure 1).

### 2.5. Blood and Kidney Sampling

At the end of the experiment, the experimental rats were anesthetized and then sacrificed for obtaining the blood samples to be analyzed. To determine both the kidney LPO and the content of kidney total thiol, glutathione, and antioxidant enzymes, kidney samples were homogenized (0.1 M) in phosphate-buffered saline (cold). Other parts from the kidney tissues were collected and put in 10% neutral buffered formalin for histological examination. Other parts of the kidney were stored at -70°C for western blotting, RNA extraction, and quantitative RT-PCR analysis.

### 2.6. Detection of the Concentrations of Serum Urea, Creatinine, and Uric Acid

The renal function parameters including urea, creatinine, and uric acid levels were estimated according to the methods of Tabacco et al. [49], Fabiny and Ertingshausen [50], and Fossati et al. [51].

### 2.7. Histological Investigation

For histological investigation, a part from the kidney of each rat was postfixed in neutral buffered formalin (10%) for twenty-four hours. After complete fixation, specimens (3-4 mm^3^) were cleared in xylene and embedded in paraffin wax, then sectioned using a microtome at a thickness of 4 *μ*m, and stained with hematoxylin and eosin (H&E) for light microscopy investigation [52].

### 2.8. Evaluation of the Kidney Oxidative Stress and Antioxidant Defense Markers

Kidney lipid peroxidation (LPO) and reduced glutathione (GSH) content were assessed using the methods of Preuss et al. [53] and Beutler et al. [54], respectively. Kidney total thiol, glutathione reductase (GR), glutathione-S-transferase (GST), glutathione peroxidase (GPx), and superoxide dismutase (SOD) were estimated using the reported methods according to Koster et al. [55], Goldberg [56], Mannervik and Gutenberg [57], Matkovics et al. [58], and Marklund and Marklund [59], respectively, with minor modifications.

### 2.9. Isolation of RNA and RT-PCR Gene Expression Analysis

The total amount of RNA from frozen kidney tissues was extracted using a Qiagen tissue extraction kit (USA) and quantified. According to the manufacturer's procedure, the concentration was estimated spectrophotometrically at A260. Two *μ*g of total RNAs were used for the reverse transcription system (Fermentas, USA). A qRT-PCR was carried out using SYBR Green mix (Thermo Fisher Scientific, USA) with PCR condition. The sequences of the primer pairs are given in Table 1. The expression of genes was determined using the 2^-*ΔΔ*Ct^ method. The *β*-actin gene was amplified as an internal control, and samples were run in triplicate.

### 2.10. Western Blot Analysis

Proteins were extracted from kidney tissue samples by RIPA buffer containing a cocktail of protease inhibitors (Bio-Rad Inc.). Protein was quantified using a Bradford assay kit (SK3041; Bio Basic Inc., Markham Ontario L3R 8T4 Canada), then separated by 10% SDS-PAGE (Bio-Rad Laboratories Inc. Cat#161-0181), and transferred to PDVF membranes. Membranes were immunoblotted with indicated primary antibodies prepared at 5% blocking buffer (1 : 1000) and were cultured overnight at 4°C. After extensive washing in TBS buffer, the membranes were incubated with the corresponding HRP-conjugated secondary antibodies (Goat anti-rabbit IgG-HRP-1 mg Goat mab-Novus Biologicals) and developed using the chemiluminescent substrate (ClarityTM Western ECL substrate Bio-Rad Cat#170-5060). *β*-Actin was used as a loading control. Image analysis software was assessed by normalization procedure of each phosphorylated active target protein versus corresponding control sample total protein on the ChemiDoc MP imager.

Primary antibodies contained antibody t-PI3K (Cat#3358), p-PI3K (sc-1637), t-Akt (Cat#9272), p-Akt (Cat#9271), t-p65 (Cat#3034), p-p65 (Cat#3033,), t-NF-*κ*B inhibitor *α* (I*κ*B*α*, Cat#9242), p-I*κ*B*α* (Cat#2859), cleaved-caspase-3 (Cat#AB3623), and *β*-actin (sc-8432).

### 2.11. Statistical Analysis

The results (mean ± SEM) obtained were performed using a one-way ANOVA test followed by Tukey's test post hoc analysis with the help of GraphPad prism 5 (GraphPad Software, CA, USA). The values of *P* > 0.05were nonsignificant while those of *P* < 0.05, *P* < 0.01, and *P* < 0.001 were presumed to be significant, highly significant, and very highly significant, respectively.

## 3. Results

### 3.1. Effects of STE, Sm, and Sb on Nephrotoxicity Biomarkers in Serum

A significant difference was observed in the renal function markers when compared in all rat groups (Table 2). The concentrations of urea, creatinine, and uric acid were significantly (*P* < 0.001) increased in the serum of DEN/AAF/CCl_4_-administered rats comparing to the normal control group. The groups supplemented with STE, Sm, and Sb from either at the 1^st^ week or the 16^th^ week of carcinogen administration revealed a significant decrease (*P* < 0.001) in the serum levels of urea, creatinine, and uric acid in comparison to the DEN/AAF/CCl_4_-administered group.

### 3.2. Effects of STE, Sm, and Sb on Kidney Histological Changes

**Figure 2:**
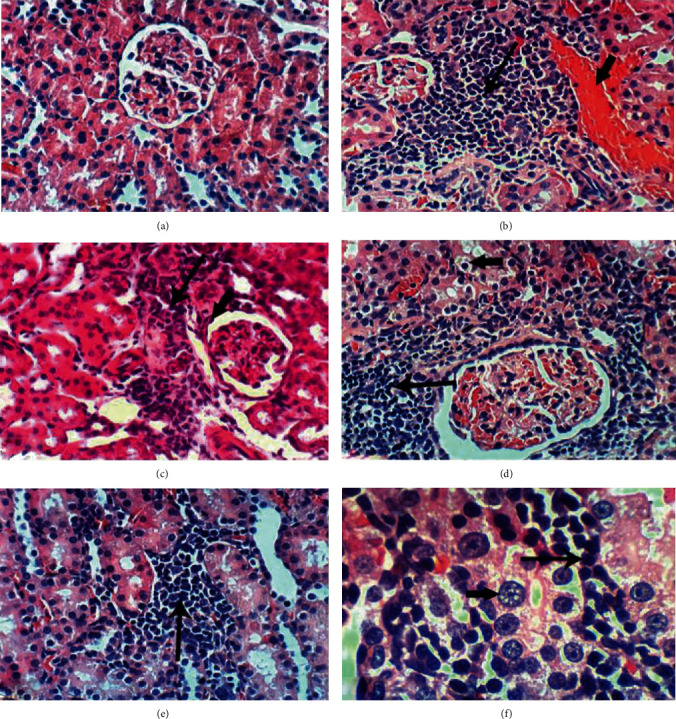
H&E-stained kidney sections photomicrographs of normal control and DEN/AAF/CCl_4_-administered rats. There were no histological changes that have been observed in normal control rats ((a); 400x). On contrary, the DEN/AAF/CCl_4_-administered rats exhibited congestion of renal blood vessel (short arrow) ((b); 400x), focal necrosis of renal tubules associated with inflammatory cell infiltration (long arrow) (b, c), thickening of the parietal layer of Bowman's capsule (short arrow) ((c); 400x), vacuolation of the renal tubular epithelium (short arrow), periglomerular inflammatory cell infiltration (long arrow) and congestion of glomerular tuft ((d); 400c), interstitial nephritis (long arrow) (e, f), and dysplastic renal tubules with karyomegalic nuclei (short arrow) ((f); 1000x).

**Figure 3:**
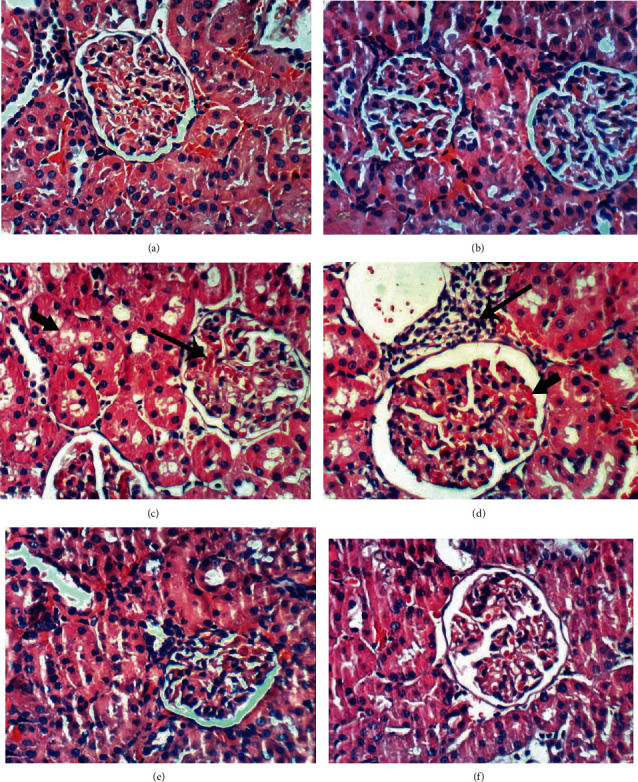
Photomicrographs (H&E; 400x) of kidney sections of DEN/AAF/CCl_4_-administered rats treated with STE, Sm, and Sb from the 1^st^ week of carcinogens-induction till the end of the experiment showing no histological changes in group-administered STE (a, b) as well as groups supplemented with Sb (e, f) in addition to vacuolation of the renal tubular epithelium (short arrow), congestion of glomerular tuft (long arrow) (c), and focal mononuclear cell infiltration (long arrow) (d) in rats treated with Sm.

**Figure 4:**
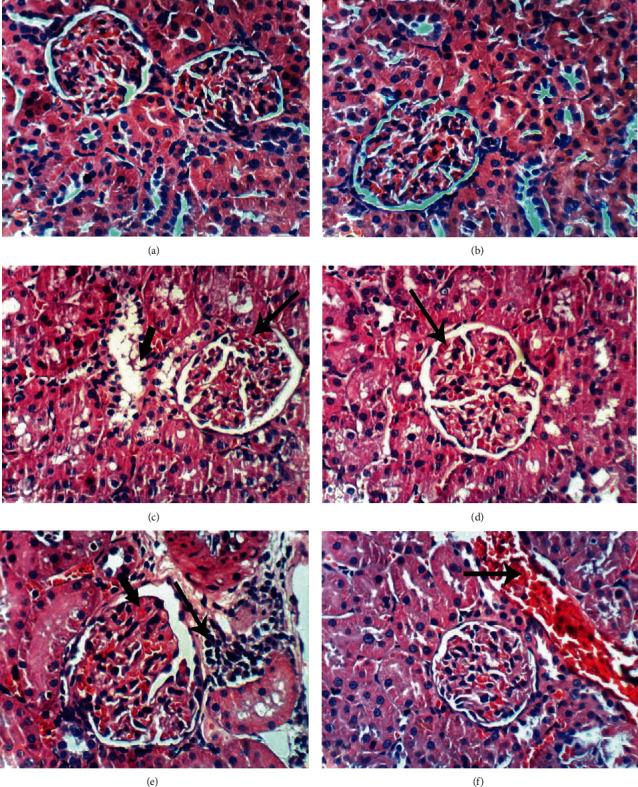
Photomicrographs of kidney sections (H&E; 400x) of DEN/AAF/CCl_4_-administered rats treated with STE, Sm, and Sb from the 16^th^ week of DEN injection till the end of the experiment. The DEN/AAF/CCl_4_-administered rats treated with STE showed no histological changes (a, b). Slight vacuolation of the renal tubular epithelium (short arrow) and congestion of glomerular tuft (long arrow) (c, d) were detected in kidney sections of rats treated with Sm. Similarly, the treatment with Sb produced a marked improvement of the kidney histological changes compared to those rats administered DEN/AAF/CCl_4_ alone; there was congestion of glomerular tuft (short arrow), focal mononuclear cell infiltration (long arrow) (e), and congestion of renal blood vessel (f).

According to H&E staining (Figures 2–4), the cells in kidney tissues of DEN/AAF/CCl_4_-administered rats showed tubular interstitial necrosis, periglomerular inflammatory cell infiltration, glomerular tuft congestion, vacuolation of epithelial lining renal tubules, interstitial nephritis, thickening of the parietal layer of Bowman's capsule, and karyomegaly of the nuclei with more than one nucleolus (Figures 2(b)–2(f)) in comparison with the intact architecture of kidney tissue of the normal control rats (Figure 2(a)), whereas the treatment with STE, Sm, and Sb from the 1^st^ week of DEN/AAF/CCl_4_ administration showed normalized kidney structure in both the STE-treated group (Figures 3(a) and 3(b)) and the Sb-treated group (Figures 3(e) and 3(f)). Furthermore, renal tubular epithelium vacuolation, glomerular tuft congestion, and focal mononuclear cell infiltration were detected in the Sm-supplemented group (Figures 3(c) and 3(d)). Similarly, the treatment with STE, Sm, and Sb from the 16^th^ week of DEN/AAF/CCl_4_ administration revealed no histological changes in STE-treated rats (Figures 4(a) and 4(b)), slight vacuolation of renal tubular epithelium and congestion of glomerular tuft in Sm-treated rats (Figures 4(c) and 4(d)), and congestion of glomerular tuft, focal mononuclear cell infiltration, and congestion of renal blood vessel in Sb-treated rats (Figures 4(e) and 4(f)).

### 3.3. Effects of STE, Sm, and Sb on Kidney Oxidative Stress and Antioxidant Defense Markers

As shown in Tables 3 and 4, DEN/AAF/CCl_4_ administration resulted in a significant increase (*P* < 0.001) in kidney LPO and a significant decrease in the GSH kidney content, in addition to a significant decline (*P* < 0.001) in total thiol content and SOD, GPx, GR, and GST activities as compared to normal control rats. Administration of STE, Sm, and Sb, either from the 1^st^ week or from the 16^th^ week of DEN administration, significantly reduced kidney LPO (*P* < 0.001) in comparison with the DEN/AAF/CCl_4_-administered group. In contrast, these treatments significantly restored (*P* < 0.001) the kidney total thiol content as well as SOD, GPx, GR, and GST activities when compared with the DEN control group. Besides, the content of kidney GSH was markedly improved either in groups administered DEN/AAF/CCl_4_ and treated with STE (*P* < 0.001), Sm (*P* < 0.05), and Sb (*P* < 0.01) from the 1^st^ week of DEN injection or in groups treated with STE (*P* < 0.01), Sm (*P* < 0.01), and Sb (*P* < 0.01) from 16^th^ week of DEN injection to the end of the experiment.

### 3.4. Effects of STE, Sm, and Sb on the Expression of the NF-*κ*B Pathway

**Figure 5:**
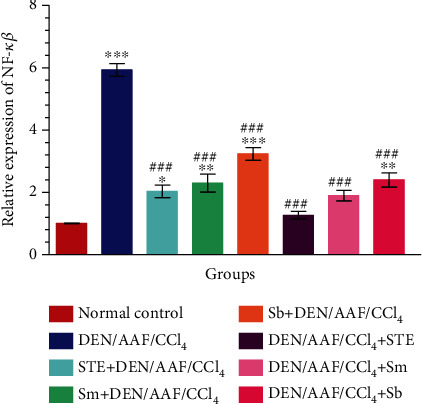
The effect of STE, Sm, and Sb on mRNA expression of NF-*κ*B of rats given DEN/AAF/CCl_4_. ^∗^*P* < 0.05, ^∗∗^*P* < 0.01, and ^∗∗∗^*P* < 0.001 were significant as compared with normal control rats, and ^#^*P* < 0.05, ^##^*P* < 0.01, and ^###^*P* < 0.001 were significant as compared with the DEN/AAF/CCl_4_-administered group. STE+DEN/AAF/CCl_4_, Sm+DEN/AAF/CCl_4_, and Sb+DEN/AAF/CCl_4_: groups orally treated with STE, Sm, and Sb, respectively, from the 1^st^ week of DEN injection; DEN/AAF/CCl_4_+STE, DEN/AAF/CCl_4_+Sm, and DEN/AAF/CCl_4_+Sb: groups orally treated with STE, Sm, and Sb, respectively, from the 16^th^ week of DEN injection.

The mRNA expression of NF-*κ*B was strongly induced in the DEN/AAF/CCl_4_-administered group when compared with the normal control group. The treatment with STE, Sm, and Sb either from the 1^st^ week or from the 16^th^ week of DEN administration reduced the NF-*κ*B expression (*P* < 0.001) to a great extent (Figure 5).

**Figure 6:**
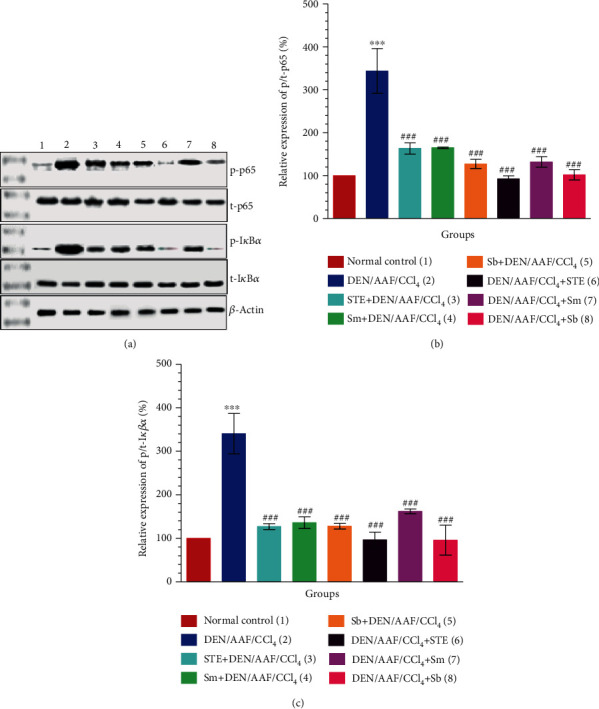
Effect of STE, Sm, and Sb on the protein levels of p65 and I*κ*B*α* in kidney tissues of rats given DEN/AAF/CCl_4_. Western blot assay was used to measure the expressions of p/t-p65 (b) and p/t-I*κ*B*α* (c) proteins in kidney tissues of the experimental groups. Representative immunoblots for quantification of p/t-p65 and p/t-I*κ*B*α* proteins were depicted in (a). Data are presented as mean values ± SEM with results from 3 independent biological repeats. ^∗^*P* < 0.05, ^∗∗^*P* < 0.01, and ^∗∗∗^*P* < 0.001 were significant as compared with normal control rats, and ^#^*P* < 0.05, ^#^#*P* < 0.01, and ^###^*P* < 0.001 were significant as compared with the DEN/AAF/CCl_4_-administered group. STE+DEN/AAF/CCl_4_, Sm+DEN/AAF/CCl_4_, and Sb+DEN/AAF/CCl_4_: groups orally treated with STE, Sm, and Sb, respectively, from the 1^st^ week of DEN injection; DEN/AAF/CCl_4_+STE, DEN/AAF/CCl_4_+Sm, and DEN/AAF/CCl_4_+Sb: groups orally treated with STE, Sm, and Sb, respectively, from the 16^th^ week of DEN injection.

The administration of DEN/AAF/CCl_4_ activated the protein levels of p/t-p65 (Figures 6(a) and 6(b); *P* < 0.001) and p/t-I*κ*B*α* (Figure 6(c); *P* < 0.001) comparing to normal control rats, whereas the groups treated with STE, Sm, and Sb either from the 1^st^ week or from the 16^th^ week of carcinogen administration showed a significantly declined ratio of p/t-p65 (Figures 6(a) and 6(b); *P* < 0.001). Similarly, the rats administered DEN/AAF/CCl_4_ then treated with STE, Sm, and sb showed a significant decline in the level of p/t-I*κ*B*α* (Figures 6(a) and 6(c); *P* < 0.001) manifesting that the STE, Sm, and Sb restrained NF-*κ*B signaling pathways.

**Figure 7:**
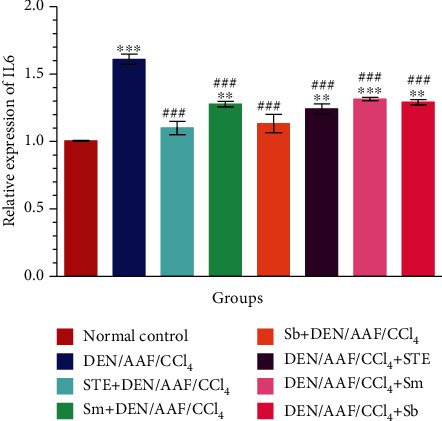
The effect of STE, Sm, and Sb on mRNA expression of IL-6 of rats given DEN/AAF/CC_4_. ^∗^*P* < 0.05, ^∗∗^*P* < 0.01, and ^∗∗∗^*P* < 0.001 were significant as compared with normal control rats, and ^#^*P* < 0.05, ^##^*P* < 0.01, and ^###^*P* < 0.001 were significant as compared with the DEN/AAF/CCl_4_-administered group. STE+DEN/AAF/CCl_4_, Sm+DEN/AAF/CCl_4_, and Sb+DEN/AAF/CCl_4_: groups orally treated with STE, Sm, and Sb, respectively, from the 1^st^ week of DEN injection; DEN/AAF/CCl_4_+STE, DEN/AAF/CCl_4_+Sm, and DEN/AAF/CCl_4_+Sb: groups orally treated with STE, Sm, and Sb, respectively, from the 16^th^ week of DEN injection.

As illustrated in Figure 7, the administration of DEN/AAF/CCl_4_ significantly (*P* < 0.001) increased the mRNA expression of IL-6 compared to normal control rats. On contrary, the treatment with STE, Sm, and Sb either from the 1^st^ week or from the 16^th^ week of carcinogen administration suppressed the expression (*P* < 0.001) of IL-6 when compared with the DEN/AAF/CCl_4_-administered group.

### 3.5. Effect of STE, Sm, and Sb on the Expression of Nrf2 in Rats Administered DEN/AAF/CCl_4_

**Figure 8:**
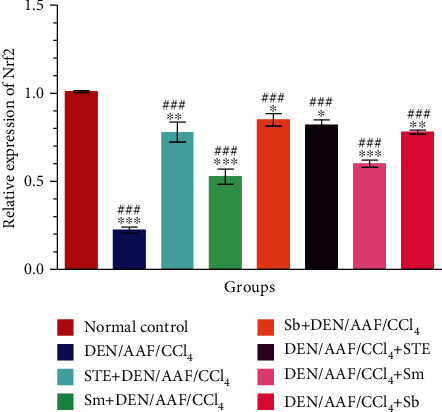
The effect of STE, Sm, and Sb on mRNA expression of Nrf2 of rats given DEN/AAF/CCl_4_. ^∗^*P* < 0.05, ^∗∗^*P* < 0.01, and ^∗∗∗^*P* < 0.001 were significant as compared with normal control rats, and ^#^*P* < 0.05, ^##^*P* < 0.01, and ^###^*P* < 0.001 were significant as compared with the DEN/AAF/CCl_4_-administered group. STE+DEN/AAF/CCl_4_, Sm+DEN/AAF/CCl_4_, and Sb+DEN/AAF/CCl_4_: groups orally treated with STE, Sm, and Sb, respectively, from the 1^st^ week of DEN injection; DEN/AAF/CCl_4_+STE, DEN/AAF/CCl_4_+Sm, and DEN/AAF/CCl_4_+Sb: groups orally treated with STE, Sm, and Sb, respectively, from the 16^th^ week of DEN injection.

As shown in Figure 8, DEN/AAF/CCl_4_ induction resulted in a marked decline in the mRNA expression of Nrf2 (*P* < 0.001) when compared with normal control rats. On contrary, in comparison with the DEN/AAF/CCl_4_-administered group, STE, Sm, and Sb supplementation either from the 1^st^ week or from the 16^th^ week of DEN administration activated this defense pathway to a great extent.

### 3.6. Effects of STE, Sm, and Sb on the mRNA Expressions of PPAR*γ* and IL-4

**Figure 9:**
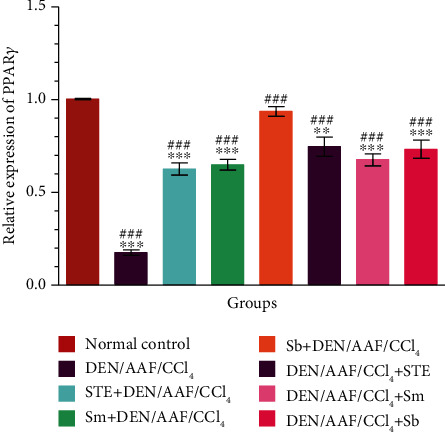
The effect of STE, Sm, and Sb on mRNA expression of PPAR*γ* of rats given DEN/AAF/CCl_4_. ^∗^*P* < 0.05, ^∗∗^*P* < 0.01, and ^∗∗∗^*P* < 0.001 were significant as compared with normal control rats, and ^#^*P* < 0.05, ^##^*P* < 0.01, and ^###^*P* < 0.001 were significant as compared with the DEN/AAF/CCl_4_-administered group. STE+DEN/AAF/CCl_4_, Sm+DEN/AAF/CCl_4_, and Sb+DEN/AAF/CCl_4_: groups orally treated with STE, Sm, and Sb, respectively, from the 1^st^ week of DEN injection; DEN/AAF/CCl_4_+STE, DEN/AAF/CCl_4_+Sm, and DEN/AAF/CCl_4_+Sb: groups orally treated with STE, Sm, and Sb, respectively, from the 16^th^ week of DEN injection.

DEN/AAF/CCl_4_ administration significantly (*P* < 0.001) decreased the expression of PPAR*γ* comparing to the normal control group, whereas supplementation of STE, Sm, and Sb either from the 1^st^ week or from the 16^th^ week of DEN administration significantly (*P* < 0.001) ameliorated this effect by increasing the mRNA expression of PPAR*γ* (Figure 9).

**Figure 10:**
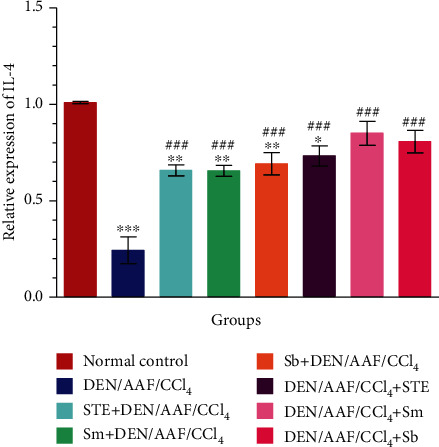
The effect of STE, Sm, and Sb on mRNA expression of IL-4 of rats given DEN/AAF/CCl_4_. ^∗^*P* < 0.05, ^∗∗^*P* < 0.01, and ^∗∗∗^*P* < 0.001 were significant as compared with normal control rats, and ^#^*P* < 0.05, ^##^*P* < 0.01, and ^###^*P* < 0.001 were significant as compared with the DEN/AAF/CCl_4_-administered group. STE+DEN/AAF/CCl_4_, Sm+DEN/AAF/CCl_4_, and Sb+DEN/AAF/CCl_4_: groups orally treated with STE, Sm, and Sb, respectively, from the 1^st^ week of DEN injection; DEN/AAF/CCl_4_+STE, DEN/AAF/CCl_4_+Sm, and DEN/AAF/CCl_4_+Sb: groups orally treated with STE, Sm, and Sb, respectively, from the 16^th^ week of DEN injection.

As depicted in Figure 10, a significant decline in the mRNA expression of IL-4 in rats administered DEN/AAF/CCl_4_ alone was detected (*P* < 0.001). On contrary, STE, Sm, and Sb supplementation either from the 1^st^ week or from the 16^th^ week of DEN administration significantly (*P* < 0.001) activated the expression of IL-4 when compared with the DEN/AAF/CCl_4_-administered group.

### 3.7. STE, Sm, and Sb Inhibit Kidney Carcinogenesis through Inhibition of the PI3K/Akt Pathway

**Figure 11:**
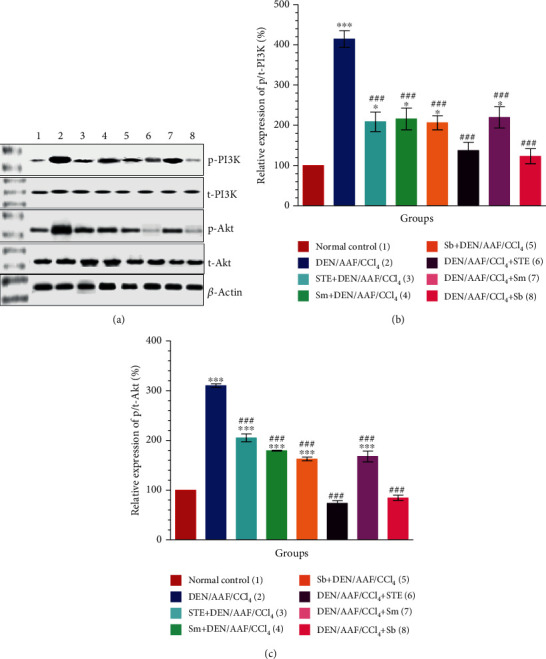
Effect of STE, Sm, and Sb on PI3K/Akt signaling pathway in kidney tissues of rats given DEN/AAF/CCl_4_. Western blot assay was used to measure the expressions of p/t-PI3K (b) and p/t-Akt (c) proteins in kidney tissues of the experimental groups. Representative immunoblots for quantification of p/t-PI3K and p/t-Akt proteins were depicted in (a). Data are presented as mean values ± SEM with results from 3 independent biological repeats. ^∗^*P* < 0.05, ^∗∗^*P* < 0.01, and ^∗∗∗^*P* < 0.001 were significant as compared with normal control rats, and ^#^*P* < 0.05, ^##^*P* < 0.01, and ^###^*P* < 0.001 were significant as compared with the DEN/AAF/CCl_4_-administered group. STE+DEN/AAF/CCl_4_, Sm+DEN/AAF/CCl_4_, and Sb+DEN/AAF/CCl_4_: groups orally treated with STE, Sm, and Sb, respectively, from the 1^st^ week of DEN injection; DEN/AAF/CCl_4_+STE, DEN/AAF/CCl_4_+Sm, and DEN/AAF/CCl_4_+Sb: groups orally treated with STE, Sm, and Sb, respectively, from the 16^th^ week of DEN injection.

Expressions of PI3K/Akt in kidney tissues of Wistar rats in the current study were assessed by western blot. As shown, the levels of p/t-PI3K (Figures 11(a) and 11(b); *P* < 0.001) and p/t-Akt (Figures 11(a) and 11(c); *P* < 0.001) were significantly increased in rats administered DEN/AAF/CCl_4_, whereas the rats treated with STE, Sm, and Sb from the 1^st^ week or the 16^th^ week of carcinogen administration showed a significant (*P* < 0.001) reduction in the protein levels of p/t-PI3K (Figures 11(a) and 11(b); *P* < 0.001). Furthermore, the protein level of p/t-Akt was significantly (Figures 11(a) and 11(c); *P* < 0.001) decreased in rats treated with STE, Sm, and Sb from either the 1^st^ week or the 16^th^ week of DEN injection.

### 3.8. STE, Sm, and Sb Ameliorate Nephrocyte Apoptosis Induced by DEN/AAF/CCl_4_

**Figure 12:**
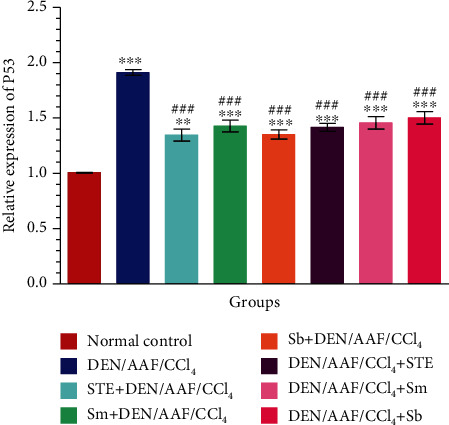
The effect of STE, Sm, and Sb on mRNA expression of p53 of rats given DEN/AAF/CCl_4_. ^∗^*P* < 0.05, ^∗∗^*P* < 0.01, and ^∗∗∗^*P* < 0.001 were significant as compared with normal control rats, and ^#^*P* < 0.05, ^##^*P* < 0.01, and ^###^*P* < 0.001 were significant as compared with the DEN/AAF/CCl_4_-administered group. STE+DEN/AAF/CCl_4_, Sm+DEN/AAF/CCl_4_, and Sb+DEN/AAF/CCl_4_: groups orally treated with STE, Sm, and Sb, respectively, from the 1^st^ week of DEN injection; DEN/AAF/CCl_4_+STE, DEN/AAF/CCl_4_+Sm, and DEN/AAF/CCl_4_+Sb: groups orally treated with STE, Sm, and Sb, respectively, from the 16^th^ week of DEN injection.

**Figure 13:**
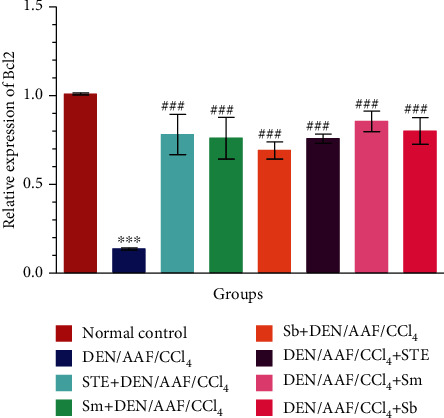
The effect of STE, Sm, and Sb on mRNA expression of Bcl-2 of rats given DEN/AAF/CCl_4_. ^∗^*P* < 0.05, ^∗∗^*P* < 0.01, and ^∗∗∗^*P* < 0.001 were significant as compared with normal control rats, and ^#^*P* < 0.05, ^##^*P* < 0.01, and ^###^*P* < 0.001 were significant as compared with the DEN/AAF/CCl_4_-administered group. STE+DEN/AAF/CCl_4_, Sm+DEN/AAF/CCl_4_, and Sb+DEN/AAF/CCl_4_: groups orally treated with STE, Sm, and Sb, respectively, from the 1^st^ week of DEN injection; DEN/AAF/CCl_4_+STE, DEN/AAF/CCl_4_+Sm, and DEN/AAF/CCl_4_+Sb: groups orally treated with STE, Sm, and Sb, respectively, from the 16^th^ week of DEN injection.

As shown in the current study, a significant (*P* < 0.001) increased mRNA expression of p53 as well as decreased expression (*P* < 0.001) of Bcl-2 in the renal tissue of rats-administered DEN/AAF/CCl_4_ was detected when compared with normal control rats. These changes were significantly (*P* < 0.001) alleviated by the coadministration of STE, Sm, and Sb from either the 1^st^ week or the 16^th^ week of DEN administration by producing low expression of p53 and strong expression of Bcl-2 comparing to DEN/AAF/CCl_4_-administered rats (Figures 12 and 13).

**Figure 14:**
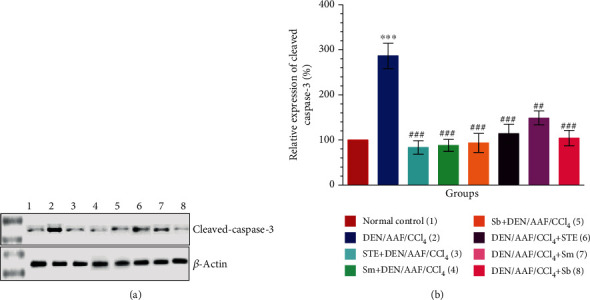
Effect of STE, Sm, and Sb on cleaved-caspase-3 in kidney tissues of rats given DEN/AAF/CCl_4_. Western blot assay was used to measure the expressions of cleaved-caspase-3 (b) protein in kidney tissues of the experimental groups. Representative immunoblots for quantification of cleaved-caspase-3 protein were depicted in (a). Data are presented as mean values ± SEM with results from 3 independent biological repeats. ^∗^*P* < 0.05, ^∗∗^*P* < 0.01, and ^∗∗∗^*P* < 0.001 were significant as compared with normal control rats, and ^#^*P* < 0.05, ^##^*P* < 0.01, and ^###^*P* < 0.001 were significant as compared with the DEN/AAF/CCl_4_-administered group. STE+DEN/AAF/CCl_4_, Sm+DEN/AAF/CCl_4_, and Sb+DEN/AAF/CCl_4_: groups orally treated with STE, Sm, and Sb, respectively, from the 1^st^ week of DEN injection; DEN/AAF/CCl_4_+STE, DEN/AAF/CCl_4_+Sm, and DEN/AAF/CCl_4_+Sb: groups orally treated with STE, Sm, and Sb, respectively, from the 16^th^ week of DEN injection.

Comparing with the normal control rats, the rats administered DEN/AAF/CCl_4_ showed a significant increment in the protein level of cleaved-caspase-3 (Figures 14(a) and 14(b); *P* < 0.001), whereas the groups treated with STE, Sm, and Sb from the 1^st^ week of carcinogen administration showed a significant (*P* < 0.001) decrease in the protein level of cleaved-caspase-3. Similarly, the level of caspase-3 was found to be reduced in rats treated with STE (*P* < 0.001), Sm (*P* < 0.01), and Sb (*P* < 0.001) starting from the 16^th^ week of DEN induction (Figures 14(a) and 14(b)).

## 4. Discussion

The kidney plays an important role in the elimination of different xenobiotics; thereby, it is more exposed to damage because of its high sensitivity to numerous drugs and toxins [60]. DEN is a well-known carcinogenic N-nitroso compound that causes preneoplastic lesions in a variety of organs in experimental animals [61, 62]. It is found mainly in agriculture products, cosmetics, alcoholic drinks, cigarette smoke, groundwater, occupational settings, dried and salted fish, foods such as cheese, and soybean [63]. Additionally, as a result of the absorption of various drugs into the body, DEN is produced in one form or repeated forms [64]. Accumulating evidence indicates that the administration of DEN in combination with AAF or CCl_4_ promotes carcinogenesis [65, 66]. When DEN is metabolized in the body by cytochrome P450, it generates highly reactive free radicals which trigger the lipid peroxidation process, oxidative damage to proteins, and DNA in other organelles of the cell [67, 68]. As a result, we investigated the preventive role of STE, Sm, and Sb against DEN-induced kidney carcinogenesis through their antioxidant property which is retained by the chemical constituents of STE, Sm, and Sb.

Urea, creatinine, and uric acid are the main breakdown products of the body processes which must be filtered and excreted by the kidneys [69]. If the kidney malfunction, these substances are not easily eliminated by them. As a result, levels of blood urea, creatinine, and uric acid will accumulate in the serum. Similarly, the administration of DEN/AAF/CCl_4_ to Wistar rats showed a significant increase in serum levels of urea, creatinine, and uric acid. Upgradation in serum creatinine levels is a key indicator of renal failure, and this range reflects the glomerular function [69]. The rise in the blood level of uric acid causes hyperuricemia, and it is a prognostic marker of inflammation that occurs at different parts of the body [70]. The administration of STE, Sm, and Sb showed a successful improvement in renal functions involving the serum levels of urea, creatinine, and uric acid in comparison with the rats administered DEN/AAF/CCl_4_ alone. Similar results were reported [34, 71], in which, Sm and Sb showed an improvement in renal functions by reducing the serum levels of creatinine, urea, and uric acid.

These biochemical changes were supported by the histopathological analysis of the renal tissue exposed to DEN/AAF/CCl_4_ showing dysplastic renal tubules and karyomegaly of the nuclei with more than one nucleolus as precancerous lesions. However, these pathological alterations were restored by STE, Sm, and Sb administration.

The presence of the long-chain polyunsaturated fatty acids in lipid composition makes the kidney vulnerable to damage that occurs by free radicals. The nitrosamine breakdown has been suggested as a way to generate ROS. Increased amounts of ROS induce somatic mutations in addition to neoplastic transformations [72]. A variety of processes in tumor cells including genetic instability and mutation progression, cellular proliferation, alterations in anticancer drug susceptibility, angiogenesis, and metastasis can be affected by intrinsic oxidative stress [73, 74]. The administration of DEN/AAF/CCl_4_ resulted in a significant elevation of MDA activity with a decrease in the glutathione (free thiols), and its oxidized glutathione disulfide, implying a substantial redox reaction within the cell. GSH is a nonenzymatic antioxidant that reacts directly with ROS or acts as a coenzyme or cofactor in the detoxification mechanism [75]. In comparison to the normal control group, the drop in kidney content of GSH may be attributed to the direct conjugation with DEN and its metabolites with free or protein bound-SH groups and a significant reduction in the GPx activity was observed. It was reported that the administration of DEN induced inflammation and disturbed the redox cycle in the kidney [76]. Moreover, DEN/AAF/CCl_4_ administration also decreased other antioxidant enzyme activities such as GST and GR. These results were strongly supported by previous studies which reported that DEN induction increased the level of MDA and reduced the kidney GSH content and the antioxidant enzymes such as SOD, GPx, GR, and GST [77, 78]. In contrast, STE, Sm, and Sb treatment significantly attenuated the elevated MDA level demonstrating that these treatments are effective in quenching free radicals. Furthermore, supplementation with STE, Sm, and Sb upregulated the activities of all renal antioxidant enzymes implying the nephroprotective effects of those treatments against DEN/AAF/CCl_4_-induced oxidative stress.

The redox imbalance, induced by excess production of ROS, triggers the transfer of Nrf2 from the cytoplasm to the nucleus, where it regulates the expression of its downstream proteins, involving HO-1 and SOD-1/-2 [79]. Accordingly, the administration of DEN/AAF/CCl_4_ resulted in a reduction in mRNA abundance of Nrf2 in the rat kidney tissues. On the other hand, the treatment with STE, Sm, and Sb succeeded in the activation of the production of Nrf2 which attains both activations of antioxidant machinery and inhibition of NF-*κ*B mediated proinflammatory pathways.

**Figure 15:**
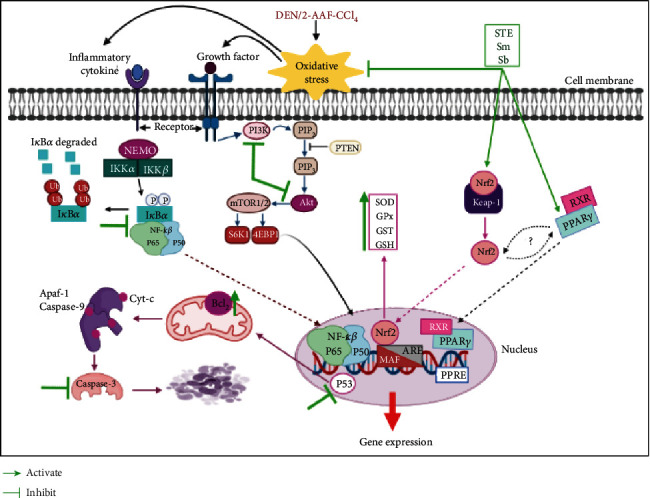
A proposed schematic diagram of the effective role of STE, Sm, and Sb on targeting NF-*κ*B, Nrf2, PPAR*γ*, PI3K/Akt, and apoptotic pathways of renal carcinogenesis.

The administration of DEN/AAF/CCl_4_ activated the expression of the proinflammatory cytokine, IL-6, which may be under the direct transcriptional directive of NF-*κ*B. This cytokine is important for inflammation, vascular permeability, and cell proliferation. Because of numerous proinflammatory cytokines, oxidative stress is also a major factor in the modulation of inflammation [80]. It also has a critical role in the development of kidney carcinoma. The redox system also plays an important role in the regulation of NF-*κ*B and various genes involved in cell transformation, proliferation, and invasion. ROS and NF-*κ*B have a complicated relationship in between. The blockage of the NF-*κ*B pathway is a good approach to control carcinogenesis and cancer progression [81]. Normally, NF-*κ*B is found in an inactive form in the cytoplasm, attached to one of a number of inhibitory molecules (I*κ*Bs), the most prevalent of which is I*κ*B*α*. I*κ*B*α* interacts with p50/p65 in the cytoplasm, forming an inactive complex. I*κ*B kinase is activated by oxidative stress which phosphorylates I*κ*B [82]. The phosphorylated I*κ*B*α* is responsible for the transfer of the active NF-*κ*B to the nucleus and interacts with *κ*B sites on target genes to promote the oncogene transcription which controls multidrug resistance, apoptosis, invasion, and metastasis [83]. It has been proved that NF-*κ*B competes with Nrf2 for transcription coactivator CREB binding protein (CBP) and can directly suppress the transcriptional level of Nrf2 [84]. Additionally, the N-terminal region of the p65 subunit of NF-*κ*B physically associates with Keap1 and inhibits the Nrf2 pathway [85]. DEN/AAF/CCl_4_ administration activated NF-*κ*B which is very delicate to ROS. STE, Sm, and Sb supplementation reduced IL-6 levels and protected against DEN/AAF/CCl_4_-induced renal carcinogenesis by inhibiting p65 and I*κ*B*α* phosphorylation, indicating further inhibition of NF-*κ*B by them (Figure 15).

Furthermore, comparing to the DEN/AAF/CCl_4_-administered group, the supplementation of STE, Sm, and Sb increased the level of IL-4. The anti-inflammatory effects of Sm and Sb have already been demonstrated in various reports against various inflammatory animal models [86, 87] which may be based on the Sm content of lipopolysaccharide, phorbol ester, ceramide, and okadaic acid [88].

The ligand-activated transcription factors, PPARs, have been revealed to play a major role in the transcription of many genes involved in various kidney physiological functions such as lipid metabolism, glucose homeostasis, and renal mineral control [89]. PPAR*γ* plays a significant role in the control of inflammation *via* suppression of the activities of many transcription factors, including NF-*κ*B and signal transducer and activator of transcription (STATs) [90, 91] involved in inflammation. PPAR*γ* is considered a tumor suppressor by controlling cell differentiation, regulation of inflammation, and its ligands may inhibit angiogenesis by downregulating the vascular endothelial growth factor (VEGF) [92]. Moreover, the interaction between Nrf2 and PPAR*γ* was investigated by various studies [25, 93] which have been proposed to suppress NF-*κ*B [94]. The current work indicated that targeting the PPAR*γ* pathway may be one of the prospective therapeutic strategies that may help in the treatment of renal cell carcinogenesis induced by DEN/AAF/CCl_4_. The present data demonstrated that the treatment of STE, Sm, and Sb significantly activated the expression of PPAR*γ* which may stop the production of downstream inflammatory mediators in the renal tissues of rat-administered DEN/AAF/CCl_4_ (Figure 15).

The PI3K-Akt signaling is permanently active in different human malignancies, and the genetic alterations of the PI3K/Akt pathway are commonly found in kidney cancer where it plays a crucial role in tumor development and therapy resistance [31, 32, 95, 96]. Our study showed that DEN/AAF/CCl_4_ exposure activated the PI3K/Akt signaling pathway. When PI3K is activated, it generates phosphoinositol triphosphate (PIP3) and other related lipid second messengers, which triggers Akt/protein kinase B (PKB). Activated Akt phosphorylates a number of cytoplasmic proteins and controls a number of important cellular functions. The mTOR protein subfamily is one of Akt's most significant downstream effectors [97]. Once mTOR is activated, it enhances proliferation and inactivates BAD which inhibits apoptosis and promotes cell survival [98]. Normally, this pathway is negatively regulated by the phosphatase and tumor suppressor phosphatase on chromosome 10 (phosphatase and tensin homolog (PTEN)) which dephosphorylates PIP3 and prevents Akt activation and functions as a negative regulator of PI3K/Akt signaling pathway [98] (Figure 15).

Taken together, there is a strong link between the PI3K/Akt signaling system and carcinogenesis, suggesting that inhibiting this pathway might be an effective kidney cancer therapy strategy. Therefore, the current study targeted this pathway as one of the most promising targets to explore safer and effective therapeutic strategies against kidney cancer. As shown above, the treatment with STE, Sm, and Sb either from the 1st week or from the 16th weeks of carcinogen induction significantly blocked the PI3K/Akt signaling pathway *via* inhibition of the protein levels of PI3K and Akt in DEN/AAF/CCl_4_-administered rats (Figure 15).

High ROS exposure increases mitochondrial permeability transition and alters transmembrane potential, resulting in mitochondrial oxidative damage and increased ROS release into the cytoplasm [99]. In conjugation with mitochondrial apoptosis, proapoptotic molecules produced from mitochondria, involving cytochrome c, trigger the release of caspase-3 and caspase-9, which cleave particular substrate proteins, such as PARP, to activate damage of DNA [100]. Meanwhile, DEN/AAF/CCl_4_ administration resulted in an imbalance in the expression of pro- and antiapoptotic Bcl-2 family members. ROS may be generated due to this imbalance and consequently activates caspase-8 and caspase-9 [101]. In a feedback loop, once caspase-8 is activated, it not only activates caspase-3 but also leads to mitochondrial apoptosis through cleaved Bid [102]. On the other hand, the treatment with STE, Sm, and Sb increased the expression of Bcl-2 while reduced the levels of p53 and cleaved-caspase-3, confirming that STE, Sm, and Sb may have an anticarcinogenic mechanism. This might be due to the modulation of oxidative stress-induced apoptosis.

According to the previous findings, STE is the most potent antikidney carcinogenic agent rather than Sm and Sb. This can be explained by the added oily part in STE which contains a higher amount of tocopherols particularly *α*tocopherol [103]. Tocopherols are one of the important antioxidants in seed oils that can regulate signal transduction, gene expressions, and cell function modification and prevent the risk of certain cancers [104].

## 5. Conclusions

Based on the above data, it can be reached to many suggestions and conclusions. First, STE, Sm, and Sb reduced oxidative stress and limited the release of free radicals through multiple mechanisms in addition to stabilization of the kidney function markers. Second, STE, Sm, and Sb regulated various transcription factors such as inhibition of proinflammatory cytokines, inhibition of PI3K/Akt signaling, activation of Nrf2 and PPAR*γ* expressions, and suppression of apoptosis. Given these findings together, the present study proposed that STE, Sm, and Sb can exert potent antirenal carcinogenic effects. To discover the accurate mechanisms of these treatments in renal carcinogenesis, more molecular and cellular investigations are recommended.

## Figures and Tables

**Table 1:** Primer sequences used in qRT-PCR analysis.

Gene	GenBank accession number	Sequence (5′–3′)
Nrf2	NM_031789.2	F: TTGTAGATGACCATGAGTCGC
		R: TGTCCTGCTGTATGCTGCTT
NF-*κ*B	NM_001276711.1	F: TACCATGCTGTTTTGGTTAC
		R: TCAAGCTACCAATGACTTTC
IL-6	NM_012589.2	F: AGTTGCCTTCTTGGGACTGA
		R: ACTGGTCTGTTGTGGGTGGT
IL-4	NM_201270.1	F: GGAACACCACGGAGAACG
		R: GCACGGAGGTACATCACG
PPAR*γ*	NM_001145367.1	F: GGACGCTGAAGAAGAGACCTG
		R: CCGGGTCCTGTCTGAGTATG
p53	NM_030989.3	F: CAGCGTGATGATGGTAAGGA
		R: CAGCGTGATGATGGTAAGGA
Bcl-2	NM_016993.1	F: GGGATGCCTTTGTGGAACTA
		R: CTCACTTGTGGCCCAGGTAT
*β*-Actin	NM_031144.3	F: AAGGATTCCTATGTGGGCGACG R: GCCTGGATAGCAACGTACATGG

**Table 2:** Effects of STE, Sm, and Sb on serum levels of urea, creatinine, and uric acid of DEN/AAF/CCl_4_-administered rats.

Groups	Urea (mg/dL)	Creatinine (mg/dL)	Uric acid (mg/dL)
Normal control	53.17 ± 3.72	0.70 ± 0.06	1.45 ± 0.10
DEN control	98.50 ± 6.87^∗∗∗^	1.12 ± 0.05^∗∗∗^	5.12 ± 0.35^∗∗∗^
STE+DEN/AAF/CCl_4_	56.50 ± 2.68^###^	0.77 ± 0.05^###^	1.87 ± 0.16^###^
Sm+DEN/AAF/CCl_4_	59.83 ± 3.56^###^	0.82 ± 0.03^###^	2.93 ± 0.30^∗∗∗^^###^
Sb+DEN/AAF/CCl_4_	59.00 ± 3.42^###^	0.80 ± 0.06^###^	2.64 ± 0.19^∗∗^^###^
DEN/AAF/CCl_4_+STE	63.00 ± 1.90^###^	0.78 ± 0.03^###^	2.43 ± 0.12^∗^^###^
DEN/AAF/CCl_4_+Sm	45.00 ± 1.92^###^	0.80 ± 0.03^###^	2.50 ± 0.16^∗^^###^
DEN/AAF/CCl_4_+Sb	69.17 ± 2.59^###^	0.78 ± 0.04^###^	2.79 ± 0.10^∗∗∗^^###^

Data are presented as mean values ± SEM. The number of detected rats is six. ^∗^*P* < 0.05, ^∗∗^*P* < 0.01, and ^∗∗∗^*P* < 0.001 were significant as compared with normal control rats, and ^#^*P* < 0.05, ^##^*P* < 0.01, and ^###^*P* < 0.001 were significant as compared with the DEN/AAF/CCl_4_-administered group. STE+DEN/AAF/CCl_4_, Sm+DEN/AAF/CCl_4_, and Sb+DEN/AAF/CCl_4_: groups orally treated with STE, Sm, and Sb, respectively, from the 1^st^ week of DEN injection; DEN/AAF/CCl_4_+STE, DEN/AAF/CCl_4_+Sm, and DEN/AAF/CCl_4_+Sb: groups orally treated with STE, Sm, and Sb, respectively, from the 16^th^ week of DEN injection.

**Table 3:** Effects of STE, Sm, and Sb on kidney LPO, SOD, and GPx activities in DEN/AAF/CCl_4_-administered rats.

Groups	LPO (nmol MDA/100mg tissue/hour)	SOD (U/g tissue)	GPx (mU/100mg tissue)
Normal control	13.78 ± 1.09	114.30 ± 4.70	165.30 ± 2.39
DEN/AAF/CCl_4_	22.53 ± 0.99^∗∗∗^	70.99 ± 2.80^∗∗∗^	133.10 ± 1.91^∗∗∗^
STE+DEN/AAF/CCl_4_	14.47 ± 0.63^###^	112.30 ± 7.31^###^	152.90 ± 2.13^∗∗^^###^
Sm+DEN/AAF/CCl_4_	13.00 ± 0.34^###^	110.60 ± 5.27^###^	153.60 ± 1.85^∗∗^^###^
Sb+DEN/AAF/CCl_4_	11.70 ± 0.33^###^	115.10 ± 7.07^###^	153.80 ± 1.84^∗∗^^###^
DEN/AAF/CCl_4_+STE	11.82 ± 1.14^###^	120.70 ± 2.97^###^	155.10 ± 2.03^∗^^###^
DEN/AAF/CCl_4_+Sm	13.46 ± 0.42^###^	113.40 ± 6.02^###^	150.60 ± 1.54^∗∗∗^^###^
DEN/AAF/CCl_4_+Sb	15.45 ± 0.40^###^	116.00 ± 1.52^###^	155.50 ± 1.48^∗^^###^

Data are presented as mean values ± SEM. The number of detected rats is six. ^∗^*P* < 0.05, ^∗∗^*P* < 0.01, and ^∗∗∗^*P* < 0.001 were significant as compared with normal control rats, and ^#^*P* < 0.05, ^##^*P* < 0.01, and ^###^*P* < 0.001 were significant as compared with the DEN/AAF/CCl_4_-administered group. STE+DEN/AAF/CCl_4_, Sm+DEN/AAF/CCl_4_, and Sb+DEN/AAF/CCl_4_: groups orally treated with STE, Sm, and Sb, respectively, from the 1^st^ week of DEN injection; DEN/AAF/CCl_4_+STE, DEN/AAF/CCl_4_+Sm, and DEN/AAF/CCl_4_+Sb: groups orally treated with STE, Sm, and Sb, respectively, from the 16^th^ week of DEN injection.

**Table 4:** Effects of STE, Sm, and Sb on kidney total thiol and GSH contents as well as GR and GST activities in DEN/AAF/CCl_4_-administered rats.

Groups	Total thiol (nmole/100mg tissue)	GSH (nmole/100mg tissue)	GR (mU/100mg tissue)	GST (U/100mg tissue)
Normal control	217.40 ± 11.43	62.51 ± 4.55	307.60 ± 9.07	455.00 ± 10.83
Control DEN	116.40 ± 5.04^∗∗∗^	32.20 ± 3.34^∗∗∗^	75.44 ± 5.00^∗∗∗^	218.90 ± 18.28^∗∗∗^
STE+DEN/AAF/CCl_4_	214.60 ± 11.14^###^	57.28 ± 3.86^###^	300.50 ± 13.70^###^	431.40 ± 31.49^###^
Sm+DEN/AAF/CCl_4_	195.90 ± 12.85^###^	51.41 ± 4.01^#^	269.30 ± 13.76^###^	421.30 ± 8.14^###^
Sb+DEN/AAF/CCl_4_	200.60 ± 14.33^###^	52.83 ± 4.57^##^	299.90 ± 7.83^###^	427.90 ± 19.24^###^
DEN/AAF/CCl_4_+STE	218.10 ± 15.27^###^	54.51 ± 2.69^##^	286.70 ± 17.29^###^	424.50 ± 30.16^###^
DEN/AAF/CCl_4_+Sm	199.40 ± 13.81^###^	52.50 ± 4.01^##^	284.00 ± 16.62^###^	395.90 ± 15.24^###^
DEN/AAF/CCl_4_+Sb	204.60 ± 9.57^###^	54.77 ± 2.42^##^	295.20 ± 10.80^###^	390.10 ± 18.94^###^

Data are presented as mean values ± SEM. The number of detected rats is six. ^∗^*P* < 0.05, ^∗∗^*P* < 0.01, and ^∗∗∗^*P* < 0.001 were significant as compared with normal control rats, and ^#^*P* < 0.05, ^##^*P* < 0.01, and ^###^*P* < 0.001 were significant as compared with the DEN/AAF/CCl_4_-administered group. STE+DEN/AAF/CCl_4_, Sm+DEN/AAF/CCl_4_, and Sb+DEN/AAF/CCl_4_: groups orally treated with STE, Sm, and Sb, respectively, from the 1^st^ week of DEN injection; DEN/AAF/CCl_4_+STE, DEN/AAF/CCl_4_+Sm, and DEN/AAF/CCl_4_+Sb: groups orally treated with STE, Sm, and Sb, respectively, from the 16^th^ week of DEN injection.

## Data Availability

All datasets analyzed and described during the present study are available from the corresponding author on reasonable request.

## Supplementary Materials

Supplementary Materials Supplementary figure: original western blot for three repeats.
