# Supplementary material for: Tackling of Renal Carcinogenesis in Wistar Rats by Silybum marianum Total Extract, Silymarin, and Silibinin via Modulation of Oxidative Stress, Apoptosis, Nrf2, PPARγ, NF-κB, and PI3K/Akt Signaling Pathways
Source: Oxid Med Cell Longev. 2021 Sep 30;2021:7665169. doi: 10.1155/2021/7665169 (PMC8497111; doi:10.1155/2021/7665169)
Supplement: Supplementary Materials — Supplementary figure: original western blot for three repeats. [file 7665169.f1.docx]

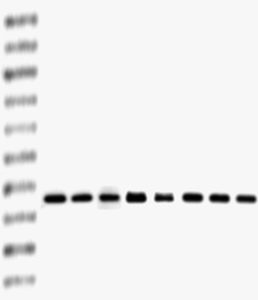

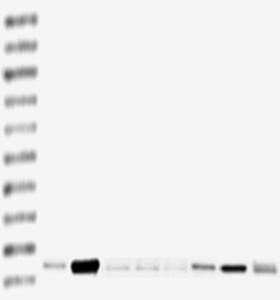

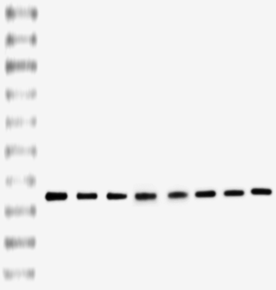

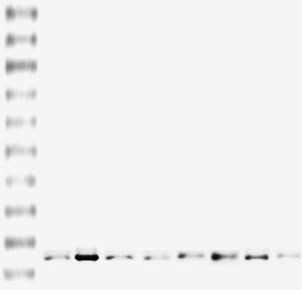

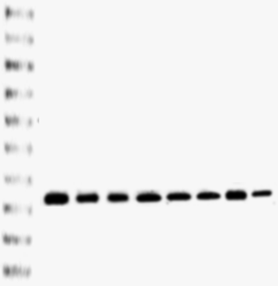

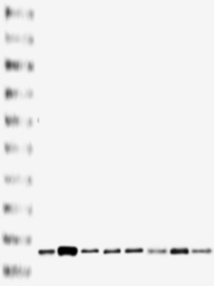

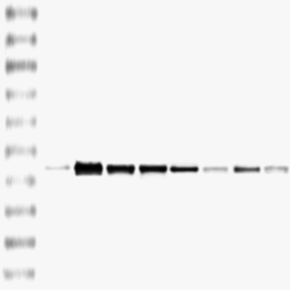

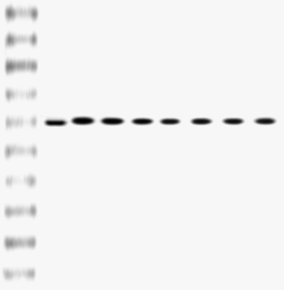

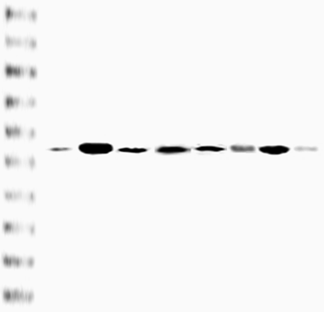

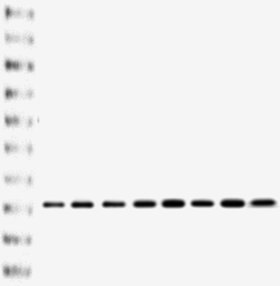

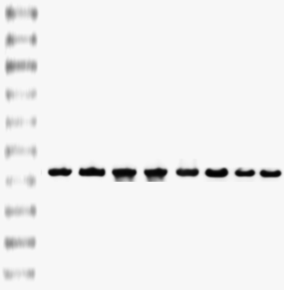

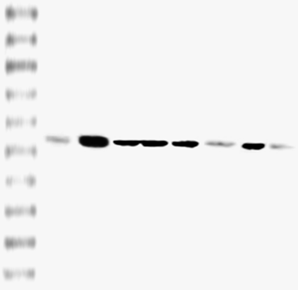

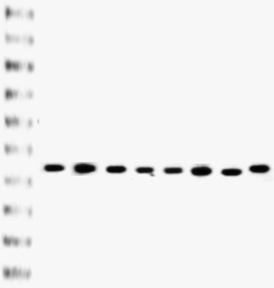

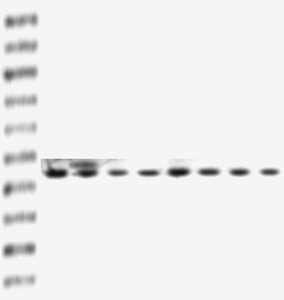

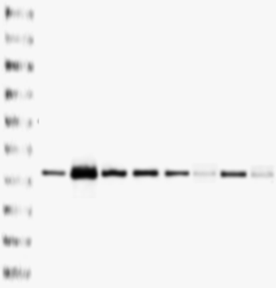

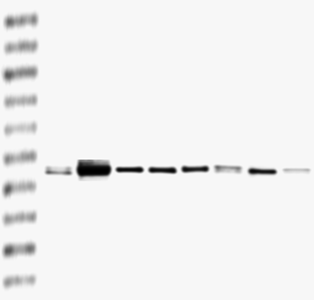

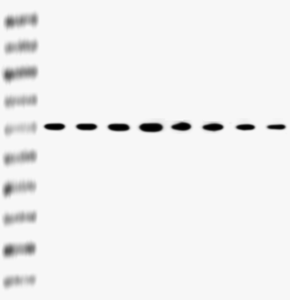

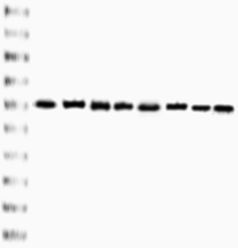

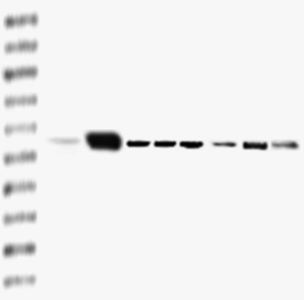

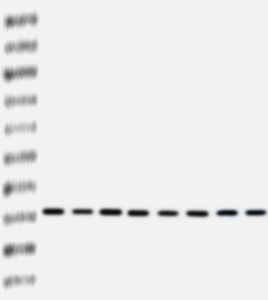

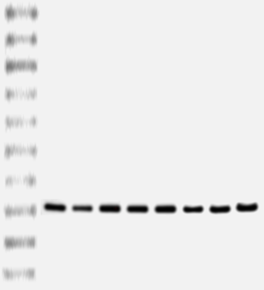

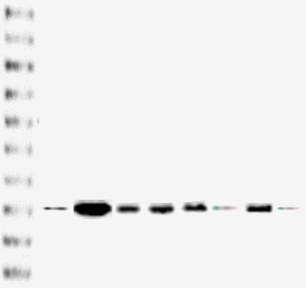

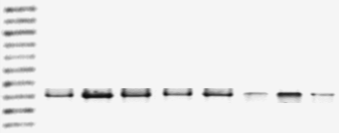

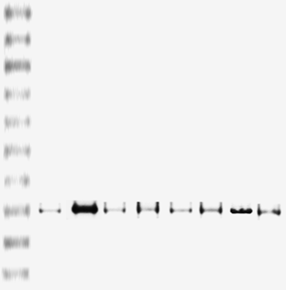

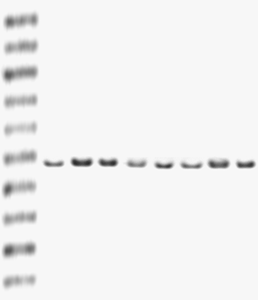

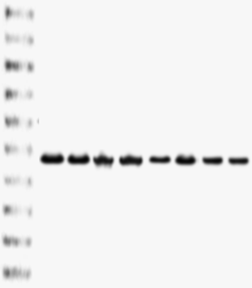

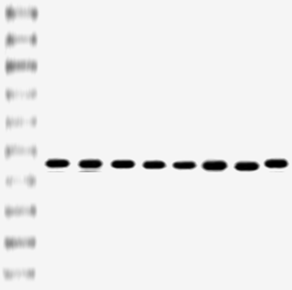

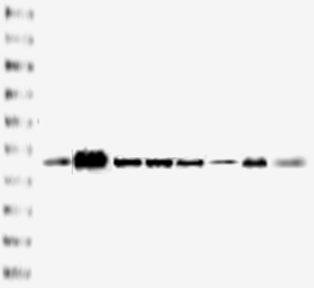

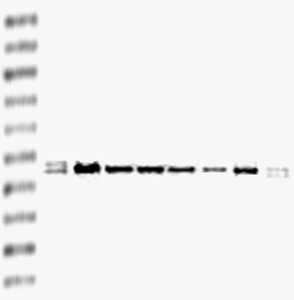

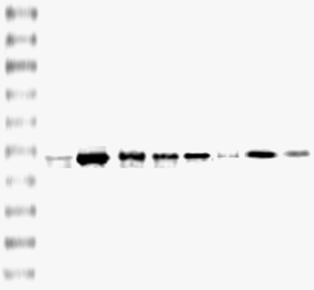


**Supplementary figure: Original western blot for three repeats.**

**Sm+DEN/AAF/CCl4**

**Normal control**

**DEN/AAF/CCl4**

**STE+DEN/AAF/CCl4**

**Sb+DEN/AAF/CCl4**

**DEN/AAF/CCl4+STE**

**DEN/AAF/CCl4+Sm**

**DEN/AAF/CCl4+Sb**

**Sm+DEN/AAF/CCl4**

**Normal control**

**DEN/AAF/CCl4**

**STE+DEN/AAF/CCl4**

**Sb+DEN/AAF/CCl4**

**DEN/AAF/CCl4+STE**

**DEN/AAF/CCl4+Sm**

**DEN/AAF/CCl4+Sb**

**Sm+DEN/AAF/CCl4**

**Normal control**

**DEN/AAF/CCl4**

**STE+DEN/AAF/CCl4**

**Sb+DEN/AAF/CCl4**

**DEN/AAF/CCl4+STE**

**DEN/AAF/CCl4+Sm**

**DEN/AAF/CCl4+Sb**

43 KDa

17 KDa

43 KDa

17 KDa

Cleaved Caspase3

17 KDa

β-actin 43 KDa

t-P65

65 KDa

p-Akt 60KDa

p-PI3K

85 KDa

t-PI3K 85 KDa

60 KDa

60 KDa

t-Akt 60 KDa

60 KDa

60 KDa

39 KDa

39 KDa

85 KDa

85 KDa

85 KDa

85 KDa

40 KDa

40 KDa

t-Iκβα 39KDa

p-Iκβα 40 KDa

65 KDa

65 KDa

65 KDa

Repeat 3

65 KDa

Repeat 2

65 KDa

p-P65

Repeat 1
